# Heavy Metal Contamination Alters the Co-Decomposition of Leaves of the Invasive Tree *Rhus typhina* L. and the Native Tree *Koelreuteria paniculata* Laxm

**DOI:** 10.3390/plants12132523

**Published:** 2023-07-01

**Authors:** Zhelun Xu, Shanshan Zhong, Youli Yu, Yue Li, Chuang Li, Zhongyi Xu, Jun Liu, Congyan Wang, Daolin Du

**Affiliations:** 1 School of Emergency Management, Jiangsu University, Zhenjiang 212013, China; xuzhelun163@163.com (Z.X.); zhongshanshan126@126.com (S.Z.); yuyoulireally@163.com (Y.Y.); liyueshine@163.com (Y.L.); lc2745144120@163.com (C.L.); llx1345631824@163.com (Z.X.); 2 School of the Environment and Safety Engineering, Jiangsu University, Zhenjiang 212013, China; 3 Jiangsu Province Engineering Research Center of Green Technology and Contigency Management for Emerging Polluants, Jiangsu University, Zhenjiang 212013, China; 4 Zhenjiang Environmental Monitoring Center of Jiangsu Province, Zhenjiang 212009, China; liujunq8@163.com; 5 Jiangsu Collaborative Innovation Center of Technology and Material of Water Treatment, Suzhou University of Science and Technology, Suzhou 215009, China

**Keywords:** invasive plants, decomposition rate, soil bacterial diversity, soil bacterial community structure, the mixed effect intensity of the co-decomposition

## Abstract

Invasive and native plants can coexist in the same habitat; however, the decomposition process may be altered by the mixing of invasive and native leaves. Heavy metal contamination may further alter the co-decomposition of both leaf types. This study evaluated the effects of two concentrations (35 mg·L^−1^ and 70 mg·L^−1^) and three types (Pb, Cu, and combined Pb + Cu) of heavy metal contamination on the co-decomposition of leaves of the invasive tree *Rhus typhina* L. and the native tree *Koelreuteria paniculata* Laxm, as well as the mixed effect intensity of the co-decomposition of the mixed leaves. A polyethylene litterbag experiment was performed over six months. The decomposition coefficient of the two trees, mixed effect intensity of the co-decomposition, soil pH and enzymatic activities, soil bacterial alpha diversity, and soil bacterial community structure were determined. A high concentration of Pb and combined Pb + Cu significantly reduced the decomposition rate of *R. typhina* leaves. A high concentration of Pb or Cu significantly reduced the decomposition rate of the mixed leaves. In general, *R. typhina* leaves decomposed faster than *K. paniculata* leaves did. There were synergistic effects observed for the co-decomposition of the mixed leaves treated with combined Pb + Cu, regardless of concentration, but there were antagonistic effects observed for the co-decomposition of the mixed leaves treated with either Pb or Cu, regardless of concentration. A high concentration of Pb or Cu may increase antagonistic effects regarding the co-decomposition of mixed-leaf groups. Thus, heavy metal contamination can significantly affect the intensity of the mixed effect on the co-decomposition of heterogeneous groups of leaves.

## 1. Introduction

Invasive plants can cause a loss of biodiversity, by altering the structures and functions of native communities [1,2,3,4]. Currently, more than 500 species of invasive plants have already invaded China. This is thought to be due mainly to the wide range of habitats and climates present in the region, as well as the increasing human activities in recent decades [5,6]. In particular, *Rhus typhina* L. has a significant impact on the structure and function of native ecosystems, and is currently considered one of the most impactful invasive tree species in China [7,8,9,10]. *Rhus typhina*, which originates from North America, was introduced to China as an ornamental and green species [5,11,12,13]. At present, research on invasive plants is mainly based on studies of herbaceous ones. As a result, it has become crucial to the field of invasion ecology to clarify the mechanisms whereby woody invasive plants such as *R. typhina* achieve successful colonization in new regions.

One of the important factors for the successful colonization of invasive plants is the potential for plant–soil interactions between invasive plants and soil microorganisms. This occurs mainly through the decomposition process [14,15,16,17], as invasive plants may benefit more from plant–soil interactions than native ones do [18,19,20,21]. Furthermore, invasive plants may produce more leaves or have their shed leaves degraded faster than native ones do, which may provide more nutrient substrate for soil micro-organisms (particularly decomposers) and subsequently improve their performance during the invasion process [17,22,23,24]. Therefore, it is crucial to elucidate the key mechanisms that invasive plant species use to achieve successful colonization, based on plant–soil interactions via the decomposition process.

Currently, most regions of China are threatened by heavy metal contamination, mainly due to the development of heavy industries [25,26,27,28]. Two types of metals, Cu and Pb, may represent key co-contaminants [25,26,27,28]. However, heavy metal contamination may alter the plant–soil interactions throughout the decomposition process, thereby affecting the invasion process of invasive plants [16,29,30,31]. Therefore, there is an urgent need to investigate the decomposition process under the co-pollution conditions involving these two metals, to elucidate the mechanisms behind the successful colonization of invasive plants, particularly woody ones. However, progress in this area is limited at present.

This study estimated the effects of two concentrations (35 mg·L^−1^ and 70 mg·L^−1^) and three types (Pb, Cu, and combined Pb + Cu) of heavy metal contamination on the co-decomposition of leaves of the invasive *R. typhina* and the native *Koelreuteria paniculata* Laxm. tree species, as well as enzymatic activities, bacterial alpha diversity, and bacterial community structures in the surrounding soil. In many parts of China, both trees are used for ecological greenery and horticultural ornamentals. They share similar habitats, and the two trees can coexist in the same area [32]. More importantly, the regions where the two trees live have been affected by severe heavy metal contamination, including Pb and Cu co-pollution [25,26,27,28]. Pb and Cu carry both environmental and ecological risks. They can decrease plant growth, as well as enhance the growth competitiveness and the allelopathy of invasive plants [33,34,35,36]. They represent the two main types of metals found in excess concentrations on arable land sites throughout China [37,38]. Pb and Cu are two of the more widely polluting metals in China, and the approximate actual soil contamination values of Pb^2+^ and Cu^2+^ in Zhenjiang, South Jiangsu, have been found to be similar (≈30–36 mg·L^−1^) [25,26,27,33,39].

This study tested the following hypotheses: (I) the decomposition rate of *R. typhina* leaves may be higher than that of *K. paniculata* leaves; (II) synergistic effects may exist regarding the co-decomposition of the mixed leaves; (III) the presence of Pb, Cu, or both, may increase the synergistic effects related to the co-decomposition of the mixed leaves.

## 2. Results

### 2.1. Differences in the Decomposition Variables

**Figure 1:**
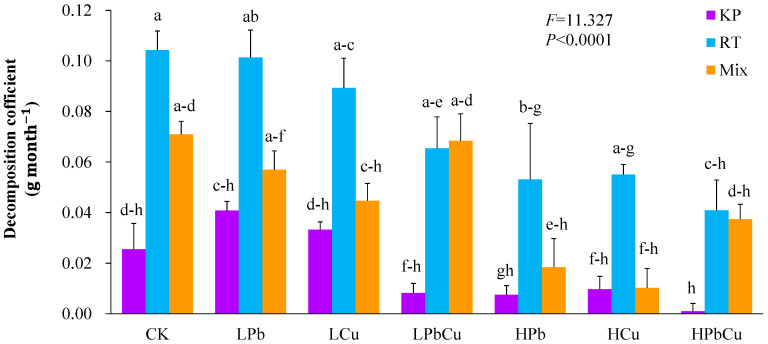
The decomposition coefficient for *Koelreuteria paniculata* Laxm (KP) and *Rhus typhina* L. (RT) leaves, and mixed leaves of this species (Mix). Bars (means and SE; *n* = 3) with different letters mean statistically significant differences (*p* < 0.05). Abbreviations: CK, control; LPb, a low concentration of Pb; LCu, a low concentration of Cu; LPbCu, a low concentration of combined Pb + Cu; HPb, a high concentration of Pb; HCu, a high concentration of Cu; HPbCu, a high concentration of combined Pb + Cu.

The *k* values of *R. typhina* leaves treated with high concentrations of Pb and combined Pb + Cu were lower than those of *R. typhina* leaves treated with the distilled water control (Figure 1; *p* < 0.05). The *k* values of the mixed leaves treated with high concentrations of Pb and Cu were lower than those of the mixture treated with the control (Figure 1; *p* < 0.05).

The *k* values of *R. typhina* leaves were higher than those of *K. paniculata* leaves, for all treatment types (Figure 1; *p* < 0.05).

The results of our three-way ANOVA analysis indicated that the type of heavy metal contamination, the type(s) of the leaves, and the interaction between the concentration of heavy metal contamination and the type(s) of the leaves significantly affected the *k* values (Table S1; *p* < 0.01).

**Figure 2:**
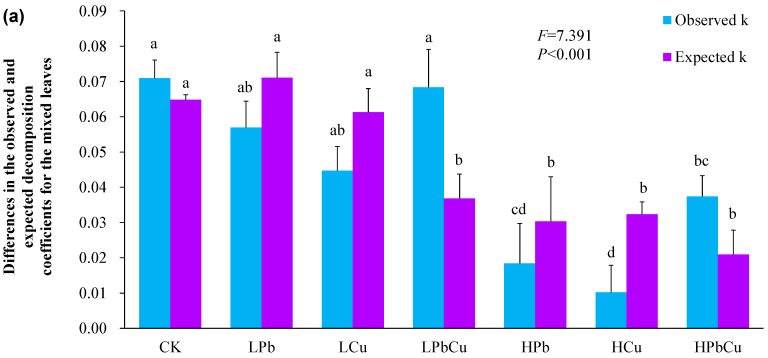

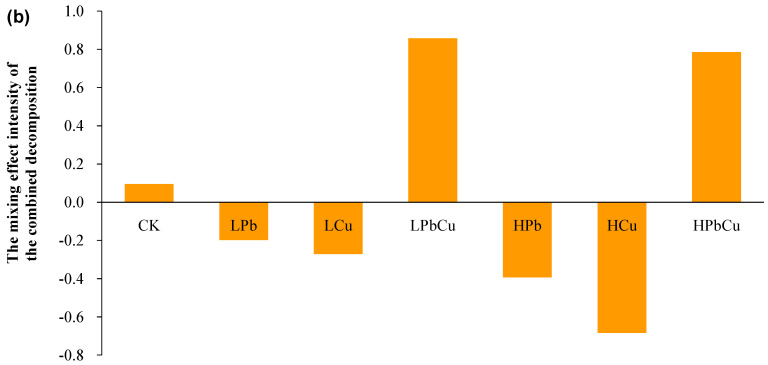
The observed (blue bars) and expected (purple bars) decomposition coefficients for the mixed *K. paniculata* and *R. typhina* leaves (**a**), and the mixing effect intensity of the co-decomposition (**b**). Bars (means and SE; *n* = 3) with different letters mean statistically significant differences (*p* < 0.05). Abbreviations have the same meanings as those presented in Figure 1.

The value of the observed *k* was higher than that of the expected *k* for the mixed leaves treated with a low concentration of combined Pb + Cu (Figure 2a; *p* < 0.05). However, the value of the observed *k* was lower than that of the expected *k* for the mixed leaves treated with high concentrations of either Pb or Cu alone (Figure 2a; *p* < 0.05).

The mixed effect intensity of the co-decomposition of leaf mixtures treated with the control and with combined Pb + Cu was higher than zero, regardless of concentration, but was lower than zero for those treated with either Pb or Cu alone, regardless of concentration (Figure 2b). The absolute value of the mixed effect intensities of co-decomposition under high concentrations of either Pb or Cu were significantly higher than those for bags treated with low concentrations of either Pb or Cu (Figure 2b).

### 2.2. Differences in Soil pH

Both heavy metal contamination and leaf type significantly increased soil pH compared to the control (Figure S1a; *p* < 0.05). The soil pHs of *K. paniculata* leaves treated with a low concentration of combined Pb + Cu, *K. paniculata* leaves treated with a high concentration of Pb, *K. paniculata* leaves treated with a high concentration of Cu, and *K. paniculata* leaves treated with a high concentration of combined Pb + Cu, were higher than that of *K. paniculata* leaves alone (Figure S1a; *p* < 0.05). Similarly, the soil pHs of *R. typhina* leaves treated with a high concentration of Pb and *R. typhina* leaves treated with a high concentration of Cu were higher than that of *R. typhina* leaves alone (Figure S1a; *p* < 0.05). The soil pH of the leaf mixture treated with a high concentration of Pb was higher than that of the leaf mixture control (Figure S1a; *p* < 0.05). Soil pHs under a high concentration of Pb, that of *K. paniculata* leaves treated with a high concentration of Pb, and that of mixed leaves treated with a high concentration of either Pb or Cu were higher than the soil pHs under a low concentration of Pb, *K. paniculata* leaves treated with a low concentration of Pb, and mixed leaves treated with a low concentration of either Pb or Cu, respectively (Figure S1a; *p* < 0.05). The soil pH of *K. paniculata* leaves treated with a low concentration of combined Pb + Cu was higher than that of *K. paniculata* leaves treated with a low concentration of Pb (Figure S1a; *p* < 0.05).

### 2.3. Differences in Soil Enzymatic Activities

Peroxidase activity levels of *K. paniculata* leaves treated with a low concentration of Pb, *R. typhina* leaves treated with a low concentration of either Pb or Cu, *K. paniculata* leaves treated with a low concentration of Cu, mixed leaves treated with a low concentration of combined Pb + Cu, mixed leaves treated with a high concentration of Pb, *R. typhina* leaves treated with a high concentration of Pb, and *K. paniculata* leaves treated with a high concentration of combined Pb + Cu were lower than those of the control (Figure S1b; *p* < 0.05). Peroxidase activity levels of *K. paniculata* leaves treated with a low concentration of Pb and *K. paniculata* leaves treated with a high concentration of combined Pb + Cu were lower than those of *K. paniculata* leaves in other conditions (Figure S1b; *p* < 0.05). The peroxidase activity level of the mixed leaves treated with a low concentration of Cu was higher than that of the mixed leaves (Figure S1b; *p* < 0.05).

Sucrase activity levels of *K. paniculata* leaves treated with a low concentration of Pb, *K. paniculata* leaves treated with a low concentration of combined Pb + Cu, and *K. paniculata* leaves treated with a high concentration of Pb were lower than those of *K. paniculata* leaves in all other conditions (Figure S1c; *p* < 0.05). The sucrase activity in the bag of *K. paniculata* leaves was higher than that that of the *R. typhina* and mixed leaf bags under the control condition (Figure S1c; *p* < 0.05).

Protease activity levels of the mixed leaves treated with a high concentration of Pb and the mixed leaves treated with a high concentration of combined Pb + Cu were lower than those of the mixed leaves treated with a high concentration of Cu (Figure S1d; *p* < 0.05).

Urease activity under a low concentration of Pb and in the mixed leaves treated with a low concentration of Pb was higher than that of the control. Urease activity levels under a high concentration of Pb and in the mixed leaves treated with a high concentration of combined Pb + Cu were lower than that of the control. The effects of Pb or Cu on urease activity were mostly concentration-dependent (Figure S1e; *p* < 0.05). The effects of Pb or Cu on urease activity were mostly concentration-dependent (Figure S1e; *p* < 0.05). Urease activity levels of *R. typhina* leaves treated with a low concentration of Pb and *R. typhina* leaves treated with a low concentration of Cu were higher than that of *R. typhina* leaves treated with a low concentration of combined Pb + Cu (Figure S1e; *p* < 0.05). Urease activity in the mixed leaves treated with a low concentration of Pb was higher than that of the mixed leaves treated with a low concentration of combined Pb + Cu (Figure S1e; *p* < 0.05). Urease activity levels under high concentrations of Cu and combined Pb + Cu were higher than that under a high concentration of Pb (Figure S1e; *p* < 0.05). Urease activity of *R. typhina* leaves treated with a high concentration of Cu was higher than that of *R. typhina* leaves treated with a high concentration of Pb and of *R. typhina* leaves treated with a high concentration of combined Pb + Cu (Figure S1e; *p* < 0.05). Urease activity of the mixed leaves treated with a high concentration of Pb was higher than that of the mixed leaves treated with a high concentration of combined Pb + Cu (Figure S1e; *p* < 0.05). Urease activity of *K. paniculata* leaves treated with a high concentration of heavy metal contamination was higher than that of *K. paniculata* leaves under other conditions (Figure S1e; *p* < 0.05). The urease activity of *R. typhina* leaves treated with a low concentration of combined Pb + Cu and *R. typhina* leaves treated with a high concentration of heavy metal contamination was higher than that of *R. typhina* leaves under other conditions (Figure S1e; *p* < 0.05). Urease activity levels of the mixed leaves treated with a low concentration of combined Pb + Cu and of the mixed leaves treated with a high concentration of heavy metal contamination were higher than that of the mixed leaves (Figure S1e; *p* < 0.05). The urease activity of *R. typhina* leaves treated with a high concentration of Cu was higher than that of *R. typhina* leaves treated with high concentrations of Pb, as well as a high concentration of combined Pb + Cu (Figure S1e; *p* < 0.05).

Phosphatase activity under a high concentration of Pb was higher than that under a high concentration of combined Pb + Cu (Figure S1f; *p* < 0.05). The phosphatase activity of *K. paniculata* leaves treated with a high concentration of Pb was lower than that of *K. paniculata* leaves under other conditions (Figure S1f; *p* < 0.05).

### 2.4. Differences in Soil Bacterial Alpha Diversity

The phylogenetic diversity indexes under a high concentration of Cu and a high concentration of combined Pb + Cu, regardless of leaf type, were higher than that of the control (Figure S2a; *p* < 0.05). The impacts of Cu and combined Pb + Cu on the phylogenetic diversity index were concentration-dependent (Figure S2a; *p* < 0.05). The phylogenetic diversity indexes under a high concentration of Cu and a high concentration of combined Pb + Cu were higher than those under a high concentration of Pb, regardless of leaf type (Figure S2a; *p* < 0.05).

The Sobs indexes of *R. typhina* leaves treated with a high concentration of Cu and a high concentration of combined Pb + Cu, and of mixed leaves treated with a high concentration of combined Pb + Cu were higher than those under the control condition (Figure S2b; *p* < 0.05). The Sobs indexes under a high concentration of Cu and for *R. typhina* leaves treated with a high concentration of Cu were higher than those under a low concentration of Cu and for *R. typhina* leaves treated with a low concentration of Cu (Figure S2b; *p* < 0.05). The Sobs index of *K. paniculata* leaves treated with a low concentration of combined Pb + Cu was higher than that of *K. paniculata* leaves treated with a low concentration of Pb (Figure S2b; *p* < 0.05). The Sobs indexes of the mixed leaves treated with a high concentration of Cu and the mixed leaves treated with a high concentration of combined Pb + Cu were higher than those of the mixed leaves treated with a high concentration of Pb (Figure S2b; *p* < 0.05).

Shannon’s diversity indexes of the mixed leaves treated with a low concentration of Pb and a high concentration of Cu were higher than those of the control (Figure S2c; *p* < 0.05).

Simpson’s dominance index under a high concentration of Cu and a high concentration of combined Pb + Cu was higher than that under the control condition (Figure S2d; *p* < 0.05). The Simpson’s dominance index under a low concentration of combined Pb + Cu was higher than that under a high concentration of combined Pb + Cu (Figure S2d; *p* < 0.05). The Simpson’s dominance index according to the type of heavy metal contamination with a high concentration was highest for a high concentration of Pb, followed by a high concentration of Cu, and finally a high concentration of combined Pb + Cu (Figure S2d; *p* < 0.05).

Pielou’s evenness indexes of *R. typhina* leaves treated with a high concentration of Pb, the mixed leaves treated with a high concentration of Pb, the mixed leaves treated with a high concentration of Cu, the mixed leaves treated with a high concentration of combined Pb + Cu, and *R. typhina* leaves treated with a high concentration of combined Pb + Cu were higher than that of the control (Figure S2e; *p* < 0.05). The Pielou’s evenness index of *R. typhina* leaves treated with a low concentration of combined Pb + Cu was higher than that of *R. typhina* leaves treated with a high concentration of combined Pb + Cu (Figure S2e; *p* < 0.05).

ACE’s richness index for the mixed leaves treated with a high concentration of combined Pb + Cu was higher than that of leaves under the control condition (Figure S2f; *p* < 0.05). ACE’s richness index for *K. paniculata* leaves treated with a low concentration of combined Pb + Cu was higher than that for *K. paniculata* leaves treated with a low concentration of Pb (Figure S2f; *p* < 0.05). The ACE’s richness index for the mixed leaves treated with a low concentration of combined Pb + Cu was higher than that for the mixed leaves treated with a low concentration of Pb (Figure S2f; *p* < 0.05). ACE’s richness index under a high concentration of combined Pb + Cu was higher than that under a low concentration of Pb, for the mixed leaves (Figure S2f; *p* < 0.05). ACE’s richness index for *K. paniculata* leaves treated with a high concentration of combined Pb + Cu was higher than that for *K. paniculata* leaves (Figure S2f; *p* < 0.05).

Chao1’s richness index for *K. paniculata* leaves treated with a low concentration of combined Pb + Cu was higher than that for *K. paniculata* leaves treated with a low concentration of Pb (Figure S2g; *p* < 0.05). Chao1’s richness index for *K. paniculata* leaves treated with a low concentration of Cu was higher than that for *K. paniculata* leaves treated with a low concentration of Pb (Figure S2g; *p* < 0.05).

The results of our three-way ANOVA analysis indicated that the concentration of heavy metal contamination significantly affected soil pH, urease activity, the phylogenetic diversity index, Sobs index, Shannon’s diversity index, Pielou’s evenness index, ACE’s richness index, and Chao1’s richness index (Table S1; *p* < 0.01). The type of heavy metal contamination significantly affected soil pH, sucrase activity, protease activity, urease activity, the phylogenetic diversity index, Sobs index, Simpson’s dominance index, Pielou’s evenness index, ACE’s richness index, and Chao1’s richness index (Table S1; *p* < 0.01). Leaf type had a significant effect on soil pH (Table S1; *p* < 0.01). The interaction of the concentration of heavy metal contamination and the type of heavy metal contamination significantly affected soil pH, peroxidase activity, protease activity, urease activity, acid phosphatase activity, and the phylogenetic diversity index (Table S1; *p* < 0.01). The interaction of the concentration of heavy metal contamination and leaf type significantly affected protease activity, urease activity, and acid phosphatase activity (Table S1; *p* < 0.01). The interaction of the type of heavy metal contamination and leaf type significantly affected urease activity, acid phosphatase activity, and Pielou’s evenness index (Table S1; *p* < 0.01). The interaction of the three factors significantly affected protease activity, urease activity, acid phosphatase activity, and Simpson’s dominance index (Table S1; *p* < 0.01).

### 2.5. Differences in Soil Bacterial Community Structure

The mean value of Good’s coverage indexes for soil bacterial communities across all samples was ≈98.47%. There were significant differences in soil bacterial beta diversity based on weighted UniFrac distances between different treatments (Figures S3–S6). The influence intensity of the concentration of heavy metal contamination and the type of heavy metal contamination on soil bacterial community structure was significantly higher than the influence intensity of leaf type (Figures S3–S6).

**Figure 3:**
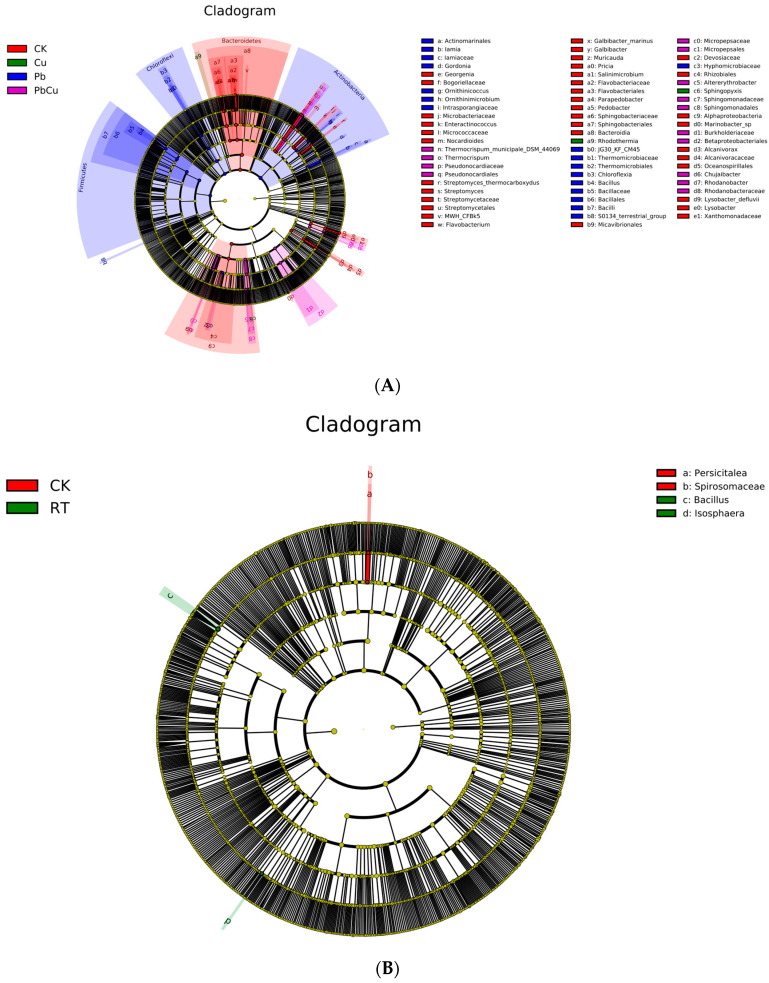
The LEfSe method identifies the significantly different abundant taxa of soil bacteria (subgraph (**A**), the type of heavy metal contamination; Subgraph (**B**), the type of the leaves). The taxa with significantly different abundances among treatments are signified by colored dots, and from the center outward, they mean the kingdom, phylum, class, order, family, genus, and species levels, respectively. The colored shadows mean trends of the significantly differed taxa. Only taxa meeting an LDA significance threshold of >2 are displayed. Abbreviations have the same meanings as those presented in Figure 1.

Under the perspective comparison among the effects of heavy metal contamination, Georgenia, *Bogoriellaceae*, *Microbacteriaceae*, *Enteractinococcus*, *Nocardioides*, *Streptomyces*_*thermocarboxydus*, *Streptomyces*, *Streptomycetaceae*, *Streptomycetales*, MWH_CFBk5, Flavobacterium, *Galbibacter*_marinus, *Galbibacter*, *Muricauda*, *Pricia*, *Salinimicrobium*, *Flavobacteriaceae*, *Flavobacteriales*, *Parapedobacter*, *Pedobacter*, *Sphingobacteriaceae*, *Sphingobacteriales*, *Bacteroidia*, *Micavibrionales*, *Devosiaceae*, *Rhizobiales*, *Alphaproteobacteria*, *Marinobacter*_sp, *Alcanivorax*, *Alcanivoracaceae*, *Oceanospirillales*, *Lysobacter_defluvii*, *Lysobacter*, and *Xanthomonadaceae* were the most altered taxa of soil bacterial taxa under the control (Figure 3A); *Actinomarinales*, *Lamia*, *Lamiaceae*, *Gordonia*, *Ornithinicoccus*, *Ornithinimicrobium*, *Intrasporangiaceae*, JG30 KF CM45, *Thermomicrobiaceae*, *Thermomicrobiales*, *Chloroflexia*, *Bacillus*, *Bacillaceae*, *Bacillales*, *Bacilli*, S0134_terrestrial_group, and *Hyphomicrobiaceae* were the most altered taxa of soil bacterial taxa under Pb treatment (Figure 3A); *Rhodothermia* and *Sphingopyxis* were the most changed taxa of soil bacterial taxa under Cu treatment (Figure 3A). *Thermocrispum*, *Pseudonocardiaceae*, *Pseudonocardiales*, *Micropepsaceae*, *Micropepsales*, *Altererythrobacter*, *Sphingomonadaceae*, *Sphingomonadales*, *Burkholderiaceae*, *Betaproteobacteriales*, *Chujaibacter*, *Rhodanobacter*, and *Rhodanobacteraceae* were the most greatly changed taxa of soil bacterial taxa under combined Pb + Cu treatment (Figure 3A).

Under the perspective comparison of the impacts of the leaves of the two trees, *Persicitalea* and *Spirosomaceae* were the most altered taxa of soil bacterial taxa under the control condition (Figure 3B); Bacillus and *Isosphaera* were the most altered taxa of soil bacterial taxa under *R. typhina* leaves (Figure 3B).

### 2.6. Contribution Intensity of Soil pH and Enzymatic Activities, and Soil Bacterial Alpha Diversity on K

**Figure 4:**
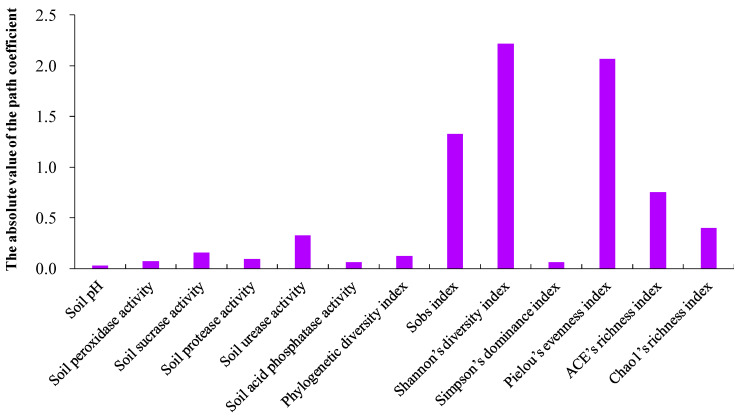
The influence intensity of soil variables and soil bacterial alpha diversity on the decomposition coefficient using the path analysis based on the absolute value of the path coefficient.

The absolute value of the direct path coefficient of the Sobs, Shannon’s diversity, Pielou’s evenness, and ACE’s richness indexes of soil bacteria was obviously larger than that of other factors (Figure 4).

## 3. Materials and Methods

### 3.1. Experimental Design

Leaves from *R. typhina* and *K. paniculata* were collected from natural sources in Zhenjiang, southern Jiangsu, China (32.205–32.216° N; 119.518–119.527° E), over the first 10 days of October 2021. Zhenjiang has a humid, northern subtropical monsoon climate. The annual mean temperature in Zhenjiang was ≈17.1 °C in 2022. The monthly mean temperature reaches a maximum of ≈28.1 °C in July and decreases to a minimum of ≈3.7 °C in January. The annual precipitation was ≈1164.1 mm in 2022, and the monthly mean precipitation reaches a maximum of ≈432.1 mm in July before dropping to a minimum of ≈2.7 mm in December. Zhenjiang received ≈1909.0 h of sunlight in 2022, and its mean monthly sunlight reaches a maximum of ≈208.2 h in December before dropping to a minimum of ≈125.9 h in August [40]. The soil type in which these two trees grow is mainly yellow soil [41]. Leaves from each tree were collected from three plant communities separated by >100 m. Leaf samples from 10 individuals of the same species were randomly collected and mixed thoroughly with other samples from the same species/community. From each individual tree, ≈50 fully expanded and intact leaves from sun-exposed parts of the plant were randomly selected, to minimize the effects of sunlight on the leaf compounds. Leaf samples were then air-dried to standardize their weights.

The decomposition process of the two trees was mimicked using a polyethylene litter bag experiment in an artificial greenhouse at Jiangsu University (located at 32.206° N; 119.512° E), under the condition of natural light, from 15 October 2021 to 15 April 2022 (experimental period: ≈6 months). The air-dried leaves of the two trees were placed in polyethylene litterbags (size: 10 × 15 cm; mesh size ≈ 0.425 mm). Leaves from the two trees were arranged in one of the following three ways per bag: 6 g of *R. typhina* leaves, 6 g of *K. paniculata* leaves, or 6 g of an equal mixture of both leaf types. The polyethylene litterbags were buried in the flower pots (upper diameter: ≈25 cm; lower diameter: ≈13 cm) which were filled with the garden soil, with one polyethylene litterbag per flowerpot. Garden soil was chosen as the culture substrate in order to maximize the possibility of an invasion history recruited by invasive plants or a pollution history mediated by metals. The garden soil was not disinfected, so as not to disturb the presence of micro-organisms (particularly the decomposers).

The polyethylene litterbags were treated with the following six types of heavy metal contaminants: a low concentration of Pb, a low concentration of Cu, a low concentration of combined Pb + Cu, a high concentration of Pb, a high concentration of Cu, and a high concentration of combined Pb + Cu, with distilled water serving as a negative control in a seventh bag. The Pb and Cu solutions were formulated using lead acetate trihydrate (purity: ≥99.0%) and copper sulfate pentahydrate (purity: ≥99.0%), respectively. The low concentration for both the independent and combined Pb^2+^ + Cu^2+^ solutions was set to 35 mg·L^−1^, to mimic the approximate actual soil contamination values of Pb^2+^ and Cu^2+^ in Zhenjiang, South Jiangsu. The high concentration for both the independent and combined Pb^2+^ and Cu^2+^ solutions was set to 70 mg·L^−1^, which greatly exceeded the common contamination level in Zhenjiang, South Jiangsu, by a large margin, in order to simulate possible future scenarios where fields are more heavily contaminated [25,26,27,33,39].

The polyethylene litterbag experiment comprised three factors: the concentration of heavy metal contamination, the type of heavy metal contamination, and the type of leaves. Each of these factors had two or three levels: two concentrations (35 mg·L^−1^ or 70 mg·L^−1^) of heavy metal contamination, three types (Pb, Cu, and combined Pb + Cu) of heavy metal contamination, and three types (*R. typhina* leaves, *K. paniculata* leaves, and the equally mixed) of the leaves of the two trees. Each treatment combination was carried out in triplicate.

After six months, all of the polyethylene litterbags were collected. Leaf samples from the two tree species were lightly scarified to remove the residual soil particles and thoroughly air-dried to standardize their weights so that decomposition variables could be more easily estimated. Soil samples were taken from within 1 cm of the polyethylene litterbags and passed through a 2 mm sieve to assess soil pH, enzymatic activities, bacterial alpha diversity, and soil bacterial community structure.

### 3.2. Determination of Decomposition Variables

The decomposition coefficient, which was used to evaluate the decomposition rate, was estimated using the following equation [42]:

(1) $$X_{t}=X_{o}*e^{-kt}$$

where *k* is the decomposition coefficient, and *X_O_* and *X_t_* denote the dry weights of the leaves at the beginning of the experiment and at time *t*, respectively. The dry weights of the leaves were measured using an electronic balance with an accuracy of 0.001 g.

The expected *k* value for the equal mixture of leaves from the two trees was evaluated as follows [43,44]:

(2) $$Expected\;k=\frac{x+y}{2}$$

where *x* and *y* denote the observed *k* values of the two trees.

The mixed effect intensity of the co-decomposition of the mixed leaves was assessed as follows [43,44]:

(3) $$The\;intensity\;of\;non-additive\;effects=\frac{O}{E}-1$$

where *O* and *E* denote the observed and expected k of the mixture of the two leaf types, respectively. Thus, intensity values greater than zero correspond to synergistic co-decomposition effects, whereas intensity values less than zero indicate antagonistic co-decomposition effects. The stronger the response, the greater the deviation from zero.

### 3.3. Determination of Soil pH and Enzymatic Activities

Soil pH was determined in situ using a digital soil acidity meter (ZD Instrument Co., Ltd., Taizhou, China).

The activities of five soil enzymes closely related to soil nutrient cycling were estimated, including (1) peroxidase (E.C. 1.11.1.1) activity—analyzed via the pyrogallol method using a colorimetric assay at 430 nm; (2) sucrase (E.C. 3.2.1.26) activity—measured Via the 3,5-dinitrosalicylic acid method with a spectrophotometer at 508 nm; (3) protease (E.C. 3.4.11.4) activity—measured using the tyrosine method with colorimetric assay at 700 nm; (4) urease (E.C. 3.5.1.5) activity—estimated via the sodium phenolate-sodium hypochlorite method with a spectrophotometer at 578 nm; and (5) acid phosphatase (E.C. 3.1.3.2) activity—estimated via the disodium phenyl phosphate method with colorimetric assay at 660 nm [45,46,47].

### 3.4. Determination of Soil Bacterial Communities

Soil bacterial communities were assessed via high-throughput sequencing using the Illumina PE250 instrument at GENE DENOVO Co., Ltd. (Guangzhou, China). The V3–V4 region of bacterial 16S rRNA genes was amplified using the universal bacterial primers 341F/806R (forward primer: 5′-CCT AYG GGR BGC ASC AG-3′; reverse primer: 5′-GGA CTA CNN GGG TAT CTA AT-3′) [48,49]. The remaining methods for determining soil bacterial communities were the same ones used in our previous related studies [15,39].

### 3.5. Statistical Analysis

Differences in the values of decomposition variables, soil pHs, soil enzymatic activity levels, and soil bacterial alpha diversity levels between the different bags were assessed using a one-way analysis of variance (ANOVA) with Tukey’s test. Three-way ANOVA was used to evaluate the effects of the concentration of heavy metal contamination, the type of heavy metal contamination, and the type of leaves, as well as their interactions with *k* values, soil pHs, soil enzymatic activities, and soil bacterial alpha diversities. The intensities of the contributions of soil pH, enzyme activities, and bacterial alpha diversity levels to *k* were evaluated using path analysis. *p* ≤ 0.05 was considered a statistically significant difference. IBM SPSS Statistics 26.0 (IBM Corp., Armonk, NY, USA) was used for all statistical analyses.

## 4. Discussion

The decomposition process is essential for nutrient cycling [14,15,16,17]. A high concentration of Pb and combined Pb + Cu significantly reduced the decomposition rate of *R. typhina* leaves (Figure 1). A high concentration of either Pb or Cu also significantly reduced the decomposition rate of mixed *R. typhina* and *K. paniculata* leaves. Thus, the nutrient cycling rates of *R. typhina* leaves and the mixed leaves may have been suppressed by high concentrations of Pb or Cu. This finding may be due to the increased energy cost of metabolism and the decreased resource utilization efficiency of soil microbial degraders under conditions with high concentrations of metals [16,50,51,52]. However, Pb or Cu did not significantly affect the rate of decomposition of *K. paniculata* leaves (Figure 1). Thus, Pb or Cu may be detrimental to the invasion of *R. typhina*, via a reduced nutrient cycling rate compared to that of *K. paniculata* alone. This may be a good thing in terms of slowing down the invasions of invasive plants that may pose threats to local ecological structures and functions—particularly with regard to biodiversity.

Consistent with the first hypothesis of our study, the decomposition rate of *R. typhina* leaves was greater than that of *K. paniculata* leaves, regardless of the addition of Pb or Cu (Figure 1). Thus, the nutrient cycling rate of *R. typhina* may be higher than that of *K. paniculate*, and is not affected by either Pb or Cu. In general, the decomposition and nutrient cycling rates of invasive plants are typically higher than those of native plants [17,53,54,55]. This may be due to the higher levels of easily degradable compounds and lower proportions of recalcitrant materials that are difficult to degrade in *R. typhina* leaves, compared to those of *K. paniculata*. Another reason why *R. typhina* leaves may have degraded faster than *K. paniculata* leaves are in this study is likely that the altered soil bacterial community structure of the leaves of the two trees, in particular *R. typhina* leaves, can trigger the emergences of certain dominant soil bacterial communities (including Bacillus and Isosphaera) (Figure 3B). Therefore, one of the main factors underlying the success of invasive species may be the faster rate of nutrient cycling mediated by a higher decomposition rate compared to that of native plants.

In any given environment, a single plant species rarely occurs alone. Usually, two or more plant species occur together (including both invasive and native plants), meaning their leaves can also coexist and decompose together [14,15,17,55]. The mixed effect intensity of the co-decomposition treated with the control, and the combined Pb + Cu conditions (regardless of concentration) was positive, but was negative when treated with Pb or Cu (also regardless of concentration). Thus, there were synergistic effects for the co-decomposition of the mixed leaves treated under the control and combined Pb + Cu conditions, regardless of concentration, but there were antagonistic effects for the co-decomposition of the mixed leaves treated with Pb or Cu, regardless of concentration. Thus, the decomposition of invasive plants can increase the decomposition of native plants [14,24,56,57] treated under the control and combined Pb + Cu conditions, regardless of concentration, but the opposite is true under Pb + Cu conditions, regardless of concentration. Thus, the type of heavy metal contamination is one of the key factors that significantly affects the intensity of the mixed effect of the co-decomposition of mixed leaves. This result is not fully consistent with the second hypothesis of our study.

The absolute value of the mixed effect intensity of co-decomposition under conditions of high concentrations of Pb or Cu was markedly higher than that under conditions of low concentrations of Pb or Cu; however, there was a similarity between the absolute value of the mixed effect intensity of co-decomposition under a low concentration of combined Pb + Cu and a high concentration of combined Pb + Cu (Figure 2b). Thus, a high concentration of Pb or Cu can intensify the antagonistic effects on the co-decomposition of the mixed leaves, compared to those under a low concentration of either Pb or Cu. However, the concentration of combined Pb + Cu did not alter the antagonistic effects on the co-decomposition of the mixed leaves. Hence, the concentration of Pb or Cu is one of the key factors that significantly affects the antagonistic effects on co-decomposition of mixed-leaf samples. This result did not fully confirm the third hypothesis of this study.

A high concentration of Pb or Cu may exert a stronger inhibitory effect on the decomposition rate and nutrient cycling rate of the co-decomposition of mixed leaves, via intensified antagonistic effects on co-decomposition—but combined Pb + Cu, regardless of concentration, may exert a positive effect on the decomposition rate and nutrient cycling rate of the co-decomposition mixed leaves, via induced synergistic effects on the co-decomposition. The main reason for the differences observed in the intensity of the mixed effect of the co-decomposition of the mixed leaves under different types of heavy metal contamination may be due to the lower diversity of microbial decomposer species in the soil under high concentrations of Pb or Cu, as well as increased species diversity in terms of soil microbial degraders under combined Pb + Cu conditions, especially at higher concentrations (Figure S2). Furthermore, different types of heavy metal contamination can cause the emergence of different dominant soil bacterial communities (Figure 3A) and changes in soil bacterial beta diversity (Figures S3–S6), as was observed in this study. The concentration and type of heavy metal contamination significantly affected the phylogenetic diversity, species number, species diversity, species evenness, and species richness of surrounding soil bacteria (Table S1). The intensity of the influence of soil bacterial alpha diversity (especially in terms of species number, diversity, and richness) on the decomposition rate was significantly greater than that of other factors, based on the results of the path analysis (Figure S2). Previous studies have also shown that soil bacterial alpha diversity is positively correlated with the decomposition rates of plant species [58,59,60].

Invasive plants can improve soil enzymatic activities through the effects of higher nutrient levels on metabolic processes [61,62,63,64]. It is therefore expected that soil enzymatic activities may be enhanced following the decomposition of *R. typhina* leaves. However, in contrast to previous studies [61,62,63,64], the decomposition of *R. typhina* leaves decreased soil sucrase activity compared to that of *K. paniculata* leaves under the control condition (Figure S1). Thus, the decomposition of *R. typhina* leaves can reduce sucrase hydrolysis capacity. The reduced soil sucrase activity with regard to the decomposition of *R. typhina* leaves may be due to the reduced levels of available nutrients in the soil subsystem and higher microbial metabolic rates. Some invasive plants (including *R. typhina*) may reduce soil enzymatic activities [14,65,66].

## 5. Conclusions

A high concentration of Pb and combined Pb + Cu significantly decreased the decomposition rate of *R. typhina* leaves. A high concentration of either Pb or Cu alone significantly decreased the decomposition rate of a mixture of *R. typhina* and *K. paniculata* leaves. However, neither Pb nor Cu had any significant effect on the decomposition rate of *K. paniculata* leaves. Therefore, Pb or Cu may be detrimental to the invasiveness of *R. typhina*, in that they may reduce the rate of nutrient cycling compared to that of *K. paniculata*. *Rhus typhina* leaves were degraded faster than *K. paniculata* leaves were. Synergistic effects were found with regard to the co-decomposition of mixed leaves treated under the control, and combined Pb + Cu conditions in this study, regardless of concentration, but there were antagonistic effects observed on the co-decomposition of mixed leaves treated with either Pb or Cu alone, regardless of concentration. The type of heavy metal contamination is one of the main factors that significantly affects the intensity of the mixed effect of the co-decomposition of the mixed leaves. A high concentration of Pb or Cu can intensify the antagonistic effects on the co-decomposition of the mixed leaves, compared to low concentrations of either Pb or Cu. The concentration of combined Pb + Cu did not alter the antagonistic effects on the co-decomposition of the mixed leaves. Thus, the concentration of Pb or Cu is one of the crucial factors that significantly affects the antagonistic effects on the co-decomposition of the mixed leaves.

## Data Availability

The datasets generated during and/or analyzed during the current study are available from the corresponding author on reasonable request.

## Supplementary Materials

The following supporting information can be downloaded at. https://www.mdpi.com/article/10.3390/plants12132523/s1. Table S1: Three-way ANOVA showing the effect of the main factors: the concentration of heavy metal contamination, the type of heavy metal contamination, and the type of the leaves, and their interactions on the decomposition coefficient (*k*), soil pH, soil enzymatic activities, and the alpha diversity of soil bacteria; Figure S1: Soil pH and soil enzymatic activities; Figure S2: Alpha diversity of soil bacteria; Figure S3: The heatmap of beta diversity estimates of soil bacteria at phylum level based on weighted UniFrac distances; Figure S4: PCA of soil bacteria based on weighted UniFrac distance; Figure S5: PCoA of soil bacteria based on weighted UniFrac distance; Figure S6: NMDS of soil bacteria based on weighted UniFrac distance.
