# Supplementary material for: Heavy Metal Contamination Alters the Co-Decomposition of Leaves of the Invasive Tree Rhus typhina L. and the Native Tree Koelreuteria paniculata Laxm
Source: Plants (Basel). 2023 Jul 1;12(13):2523. doi: 10.3390/plants12132523 (PMC10346794; doi:10.3390/plants12132523)
Supplement: Supplementary file 1 [file plants-12-02523-s001.zip › plants-2459912-supplementary.pdf]

Electronic supplementary materials:

Table S1 Three-way ANOVA showing the effect of the main factors: the concentration of heavy metal contamination (C_{HM}), the type of heavy metal contamination (T_{HM}), and the type of the leaves (T_L), and their interactions on the decomposition coefficient (*k*), soil pH, soil enzymatic activities, and the alpha diversity of soil bacteria. *P* values equal to or less than 0.05 are shown in bold.

Variable and source of variation		Type III Sum of Squares	*df*	Mean Square	*F*	*P*	Partial η^2
C_{HM}	*k*	<0.001	2	<0.001	1.638	0.207	0.072
	Soil pH	0.060	2	0.030	3.366	**0.044**	0.138
	Soil peroxidase activity	4.071	2	2.035	1.912	0.160	0.083
	Soil sucrase activity	0.257	2	0.128	1.137	0.331	0.051
	Soil protease activity	<0.001	2	<0.001	0.145	0.866	0.007
	Soil urease activity	17700.586	2	8850.293	44.371	**<0.001**	0.679
	Soil acid phosphatase activity	0.055	2	0.027	1.825	0.174	0.080
	Phylogenetic diversity index	5475.480	2	2737.740	49.064	**<0.001**	0.700
	Sobs index	202195.593	2	101097.796	47.239	**<0.001**	0.692
	Shannon's diversity index	0.630	2	0.315	19.319	**<0.001**	0.479
	Simpson's dominance index	<0.001	2	<0.001	1.992	0.149	0.087
	Pielou's evenness index	0.002	2	<0.001	8.414	**<0.001**	0.286
	ACE's richness index	314944.593	2	157472.297	44.948	**<0.001**	0.682
	Chao1's richness index	213608.857	2	106804.428	39.678	**<0.001**	0.654

T_{HM}	*k*	0.013	1	0.013	49.988	**<0.001**	0.543
	Soil pH	0.491	1	0.491	54.766	**<0.001**	0.566
	Soil peroxidase activity	3.736	1	3.736	3.509	0.068	0.077
	Soil sucrase activity	0.533	1	0.533	4.726	**0.035**	0.101
	Soil protease activity	0.011	1	0.011	6.552	**0.014**	0.135
	Soil urease activity	25563.616	1	25563.616	128.164	**<0.001**	0.753
	Soil acid phosphatase activity	<0.001	1	<0.001	0.041	0.841	<0.001
	Phylogenetic diversity index	6058.249	1	6058.249	108.572	**<0.001**	0.721
	Sobs index	113804.463	1	113804.463	53.176	**<0.001**	0.559
	Shannon's diversity index	0.006	1	0.006	0.358	0.553	0.008
	Simpson's dominance index	<0.001	1	<0.001	25.187	**<0.001**	0.375
	Pielou's evenness index	0.001	1	0.001	12.034	**0.001**	0.223
	ACE's richness index	143587.001	1	143587.001	40.985	**<0.001**	0.494
	Chao1's richness index	64238.603	1	64238.603	23.865	**<0.001**	0.362
T_L	*k*	0.032	2	0.016	63.420	**<0.001**	0.751
	Soil pH	0.086	2	0.043	4.798	**0.013**	0.186
	Soil peroxidase activity	4.153	2	2.076	1.950	0.155	0.085
	Soil sucrase activity	0.696	2	0.348	3.084	0.056	0.128
	Soil protease activity	0.003	2	0.001	0.902	0.413	0.041
	Soil urease activity	552.620	2	276.310	1.385	0.261	0.062
	Soil acid phosphatase activity	0.038	2	0.019	1.257	0.295	0.056
	Phylogenetic diversity index	198.190	2	99.095	1.776	0.182	0.078

	Sobs index	8616.289	2	4308.144	2.013	0.146	0.087
	Shannon's diversity index	0.004	2	0.002	0.110	0.896	0.005
	Simpson's dominance index	<0.001	2	<0.001	0.130	0.879	0.006
	Pielou's evenness index	<0.001	2	<0.001	0.108	0.898	0.005
	ACE's richness index	14272.340	2	7136.170	2.037	0.143	0.088
	Chao1's richness index	7334.123	2	3667.062	1.362	0.267	0.061
C_{HM} * T_{HM}	*k*	<0.001	2	<0.001	1.640	0.206	0.072
	Soil pH	0.471	2	0.236	26.286	**<0.001**	0.556
	Soil peroxidase activity	8.612	2	4.306	4.045	**0.025**	0.162
	Soil sucrase activity	0.112	2	0.056	0.497	0.612	0.023
	Soil protease activity	0.016	2	0.008	4.970	**0.012**	0.191
	Soil urease activity	2099.021	2	1049.511	5.262	**0.009**	0.200
	Soil acid phosphatase activity	0.194	2	0.097	6.456	**0.004**	0.235
	Phylogenetic diversity index	773.557	2	386.779	6.932	**0.003**	0.248
	Sobs index	3498.926	2	1749.463	0.817	0.448	0.037
	Shannon's diversity index	0.032	2	0.016	0.991	0.380	0.045
	Simpson's dominance index	<0.001	2	<0.001	0.587	0.561	0.027
	Pielou's evenness index	<0.001	2	<0.001	1.690	0.197	0.074
	ACE's richness index	2345.226	2	1172.613	0.335	0.717	0.016
	Chao1's richness index	528.457	2	264.228	0.098	0.907	0.005
C_{HM} * T_L	*k*	0.004	4	0.001	4.383	**0.005**	0.295
	Soil pH	0.022	4	0.005	0.609	0.658	0.055

	Soil peroxidase activity	4.355	4	1.089	1.023	0.407	0.089
	Soil sucrase activity	0.174	4	0.044	0.386	0.817	0.035
	Soil protease activity	0.019	4	0.005	2.918	**0.032**	0.217
	Soil urease activity	4497.093	4	1124.273	5.637	**0.001**	0.349
	Soil acid phosphatase activity	0.307	4	0.077	5.109	**0.002**	0.327
	Phylogenetic diversity index	50.035	4	12.509	0.224	0.923	0.021
	Sobs index	3647.852	4	911.963	0.426	0.789	0.039
	Shannon’s diversity index	0.053	4	0.013	0.819	0.520	0.072
	Simpson’s dominance index	<0.001	4	<0.001	2.288	0.076	0.179
	Pielou’s evenness index	<0.001	4	<0.001	1.159	0.342	0.099
	ACE’s richness index	13230.072	4	3307.518	0.944	0.448	0.082
	Chao1’s richness index	13404.763	4	3351.191	1.245	0.307	0.106
T_{HM} * T_L	*k*	<0.001	2	<0.001	1.134	0.332	0.051
	Soil pH	0.034	2	0.017	1.895	0.163	0.083
	Soil peroxidase activity	0.532	2	0.266	0.250	0.780	0.012
	Soil sucrase activity	0.161	2	0.080	0.712	0.497	0.033
	Soil protease activity	0.003	2	0.001	0.891	0.418	0.041
	Soil urease activity	3854.690	2	1927.345	9.663	**<0.001**	0.315
	Soil acid phosphatase activity	0.155	2	0.078	5.172	**0.010**	0.198
	Phylogenetic diversity index	93.986	2	46.993	0.842	0.438	0.039
	Sobs index	2224.926	2	1112.463	0.520	0.598	0.024
	Shannon’s diversity index	0.079	2	0.040	2.428	0.101	0.104

	Simpson's dominance index	<0.001	2	<0.001	1.449	0.246	0.065
	Pielou's evenness index	<0.001	2	<0.001	3.947	**0.027**	0.158
	ACE's richness index	1541.679	2	770.840	0.220	0.803	0.010
	Chao1's richness index	738.863	2	369.431	0.137	0.872	0.006
C_{HM} * T_{HM}	*k*	<0.001	4	<0.001	0.166	0.955	0.016
* T_L	Soil pH	0.016	4	0.004	0.454	0.769	0.041
	Soil peroxidase activity	5.732	4	1.433	1.346	0.269	0.114
	Soil sucrase activity	0.582	4	0.146	1.290	0.289	0.109
	Soil protease activity	0.025	4	0.006	3.948	**0.008**	0.273
	Soil urease activity	4156.099	4	1039.025	5.209	**0.002**	0.332
	Soil acid phosphatase activity	0.261	4	0.065	4.350	**0.005**	0.293
	Phylogenetic diversity index	126.871	4	31.718	0.568	0.687	0.051
	Sobs index	11935.852	4	2983.963	1.394	0.252	0.117
	Shannon's diversity index	0.138	4	0.035	2.117	0.096	0.168
	Simpson's dominance index	<0.001	4	<0.001	4.923	**0.002**	0.319
	Pielou's evenness index	<0.001	4	<0.001	2.330	0.072	0.182
	ACE's richness index	6253.077	4	1563.269	0.446	0.775	0.041
	Chao1's richness index	4154.215	4	1038.554	0.386	0.818	0.035

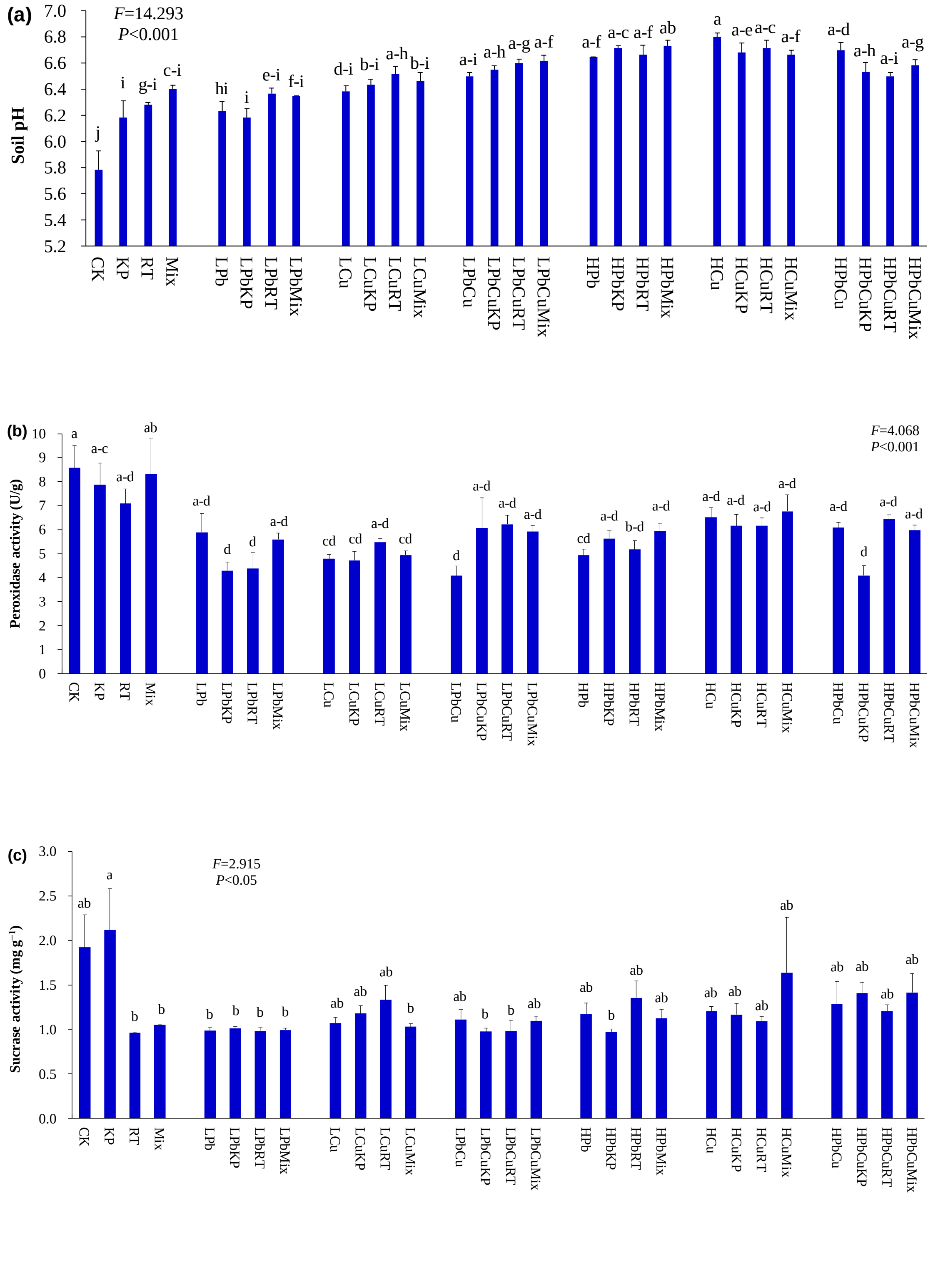
(a)
F=14.293
P<0.001
Soil pH
(b)
F=4.068
P<0.001
Peroxidase activity (U/g)
(c)
F=2.915
P<0.05
Sucrase activity (mg g−1)
CK KP RT Mix LPb LPbKP LPbRT LPbMix LCu LCuKP LCuRT LCuMix LPbCu LPbCuKP LPbCuRT LPbCuMix HPb HPbKP HPbRT HPbMix HCu HCuKP HCuRT HCuMix HPbCu HPbCuKP HPbCuRT HPbCuMix

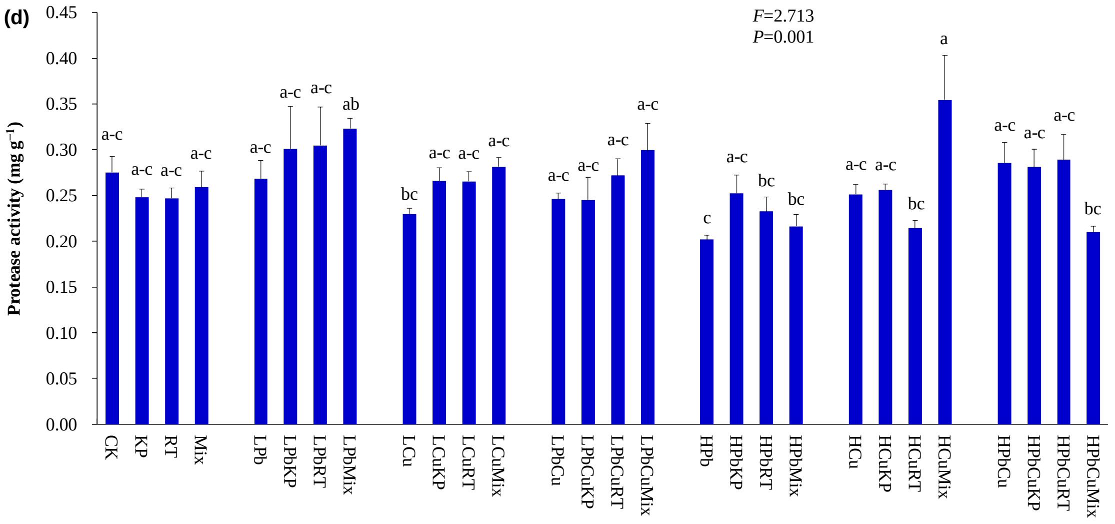

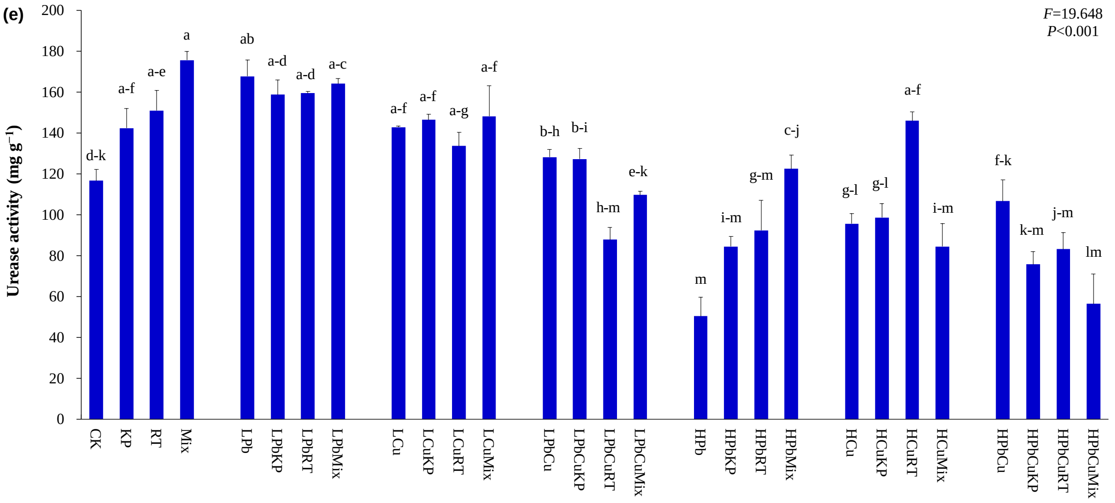

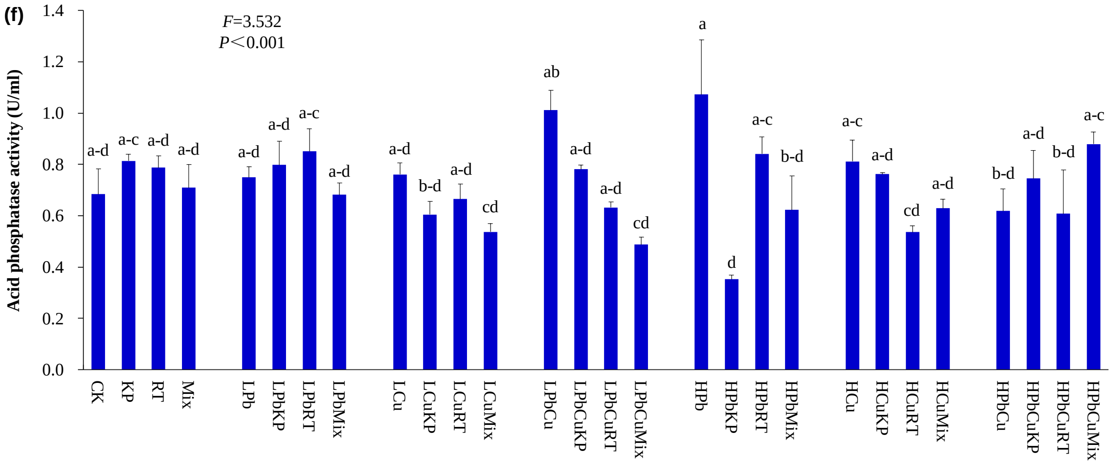

Figure S1 Soil pH and soil enzymatic activities. Bars (means and SE; n = 3) with

different letters mean statistically significant differences ($P < 0.05$). Abbreviations: KP, *K. paniculata* leaves; RT, *R. typhina* leaves; Mix, the mixed leaves of the two trees; CK, control; LPb, a low concentration of independent Pb; LCu, a low concentration of independent Cu; LPbCu, a low concentration of combined Pb + Cu; HPb, a high concentration of independent Pb; HCu, a high concentration of independent Cu; HPbCu, a high concentration of combined Pb + Cu.

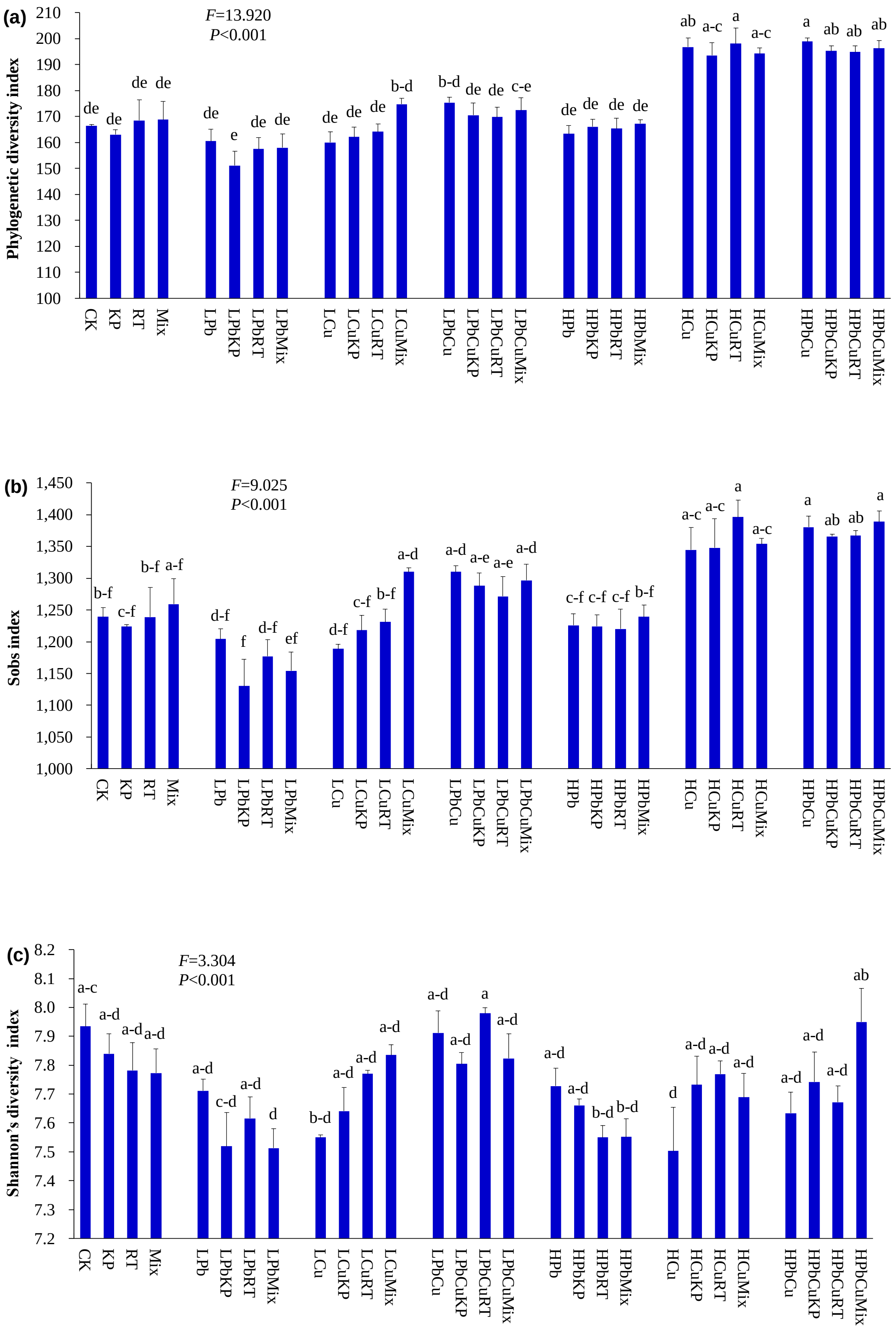
(a)
F=13.920
P<0.001
Phylogenetic diversity index
(b)
F=9.025
P<0.001
Sobs index
(c)
F=3.304
P<0.001
Shannon's diversity index
CK
KP
RT
Mix
LPb
LPbKP
LPbRT
LPbMix
LCu
LCuKP
LCuRT
LCuMix
LPbCu
LPbCuKP
LPbCuRT
LPbCuMix
HPb
HPbKP
HPbRT
HPbMix
HCu
HCuKP
HCuRT
HCuMix
HPbCu
HPbCuKP
HPbCuRT
HPbCuMix

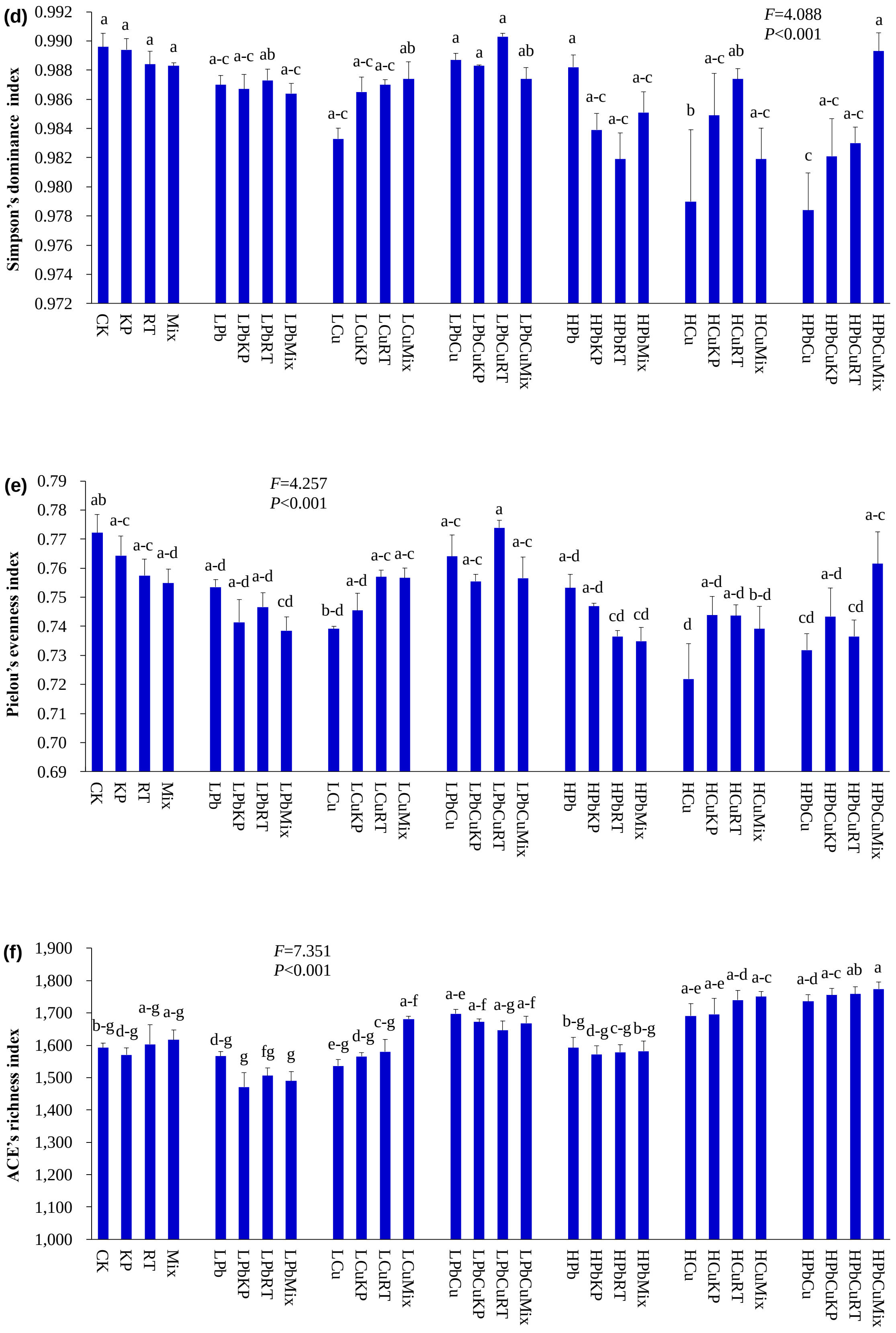
(d)
Simpson's dominance index
F=4.088
P<0.001
0.992 0.990 0.988 0.986 0.984 0.982 0.980 0.978 0.976 0.974 0.972
a a a a a-c a-c ab a-c a-c a-c a-c ab a a a ab a a-c a-c a-c b a-c ab a-c c a-c a-c a
(e)
Pielou's evenness index
F=4.257
P<0.001
0.79 0.78 0.77 0.76 0.75 0.74 0.73 0.72 0.71 0.70 0.69
ab a-c a-c a-d a-d a-d a-d cd b-d a-d a-c a-c a-c a-c a a-c a-d a-d cd cd d a-d a-d b-d cd a-d cd a-c
(f)
ACE's richness index
F=7.351
P<0.001
1,900 1,800 1,700 1,600 1,500 1,400 1,300 1,200 1,100 1,000
b-g d-g a-g a-g d-g g fg g e-g d-g c-g a-f a-e a-f a-g a-f b-g d-g c-g b-g a-e a-e a-d a-c a-d a-c ab a
CK KP RT Mix LPb LPbKP LPbRT LPbMix LCu LCuKP LCuRT LCuMix LPbCu LPbCuKP LPbCuRT LPbCuMix HPb HPbKP HPbRT HPbMix HCu HCuKP HCuRT HCuMix HPbCu HPbCuKP HPbCuRT HPbCuMix

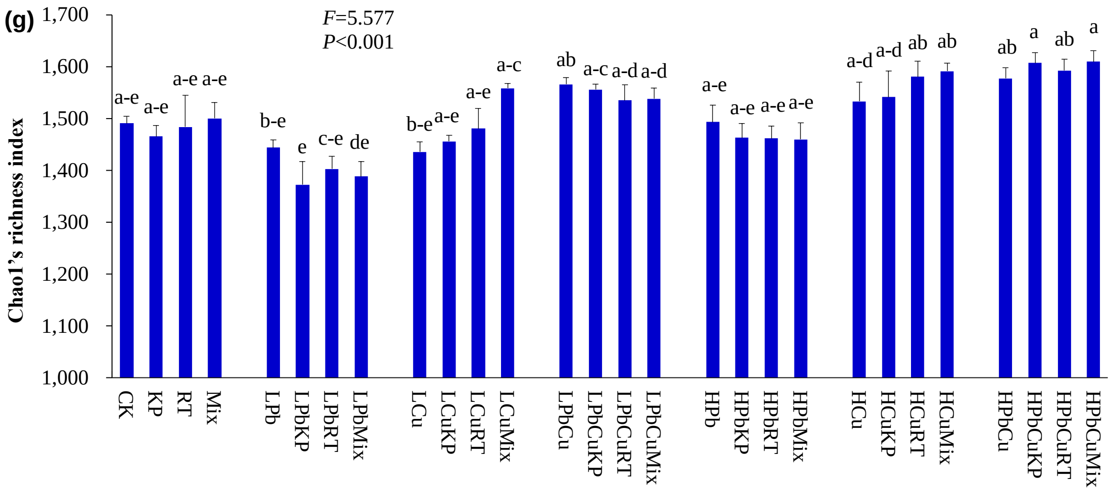

Figure S2 Alpha diversity of soil bacteria. Bars (means and SE; $n = 3$) with different letters mean statistically significant differences ($P < 0.05$). Abbreviations have the same meanings as presented in Figure S1.

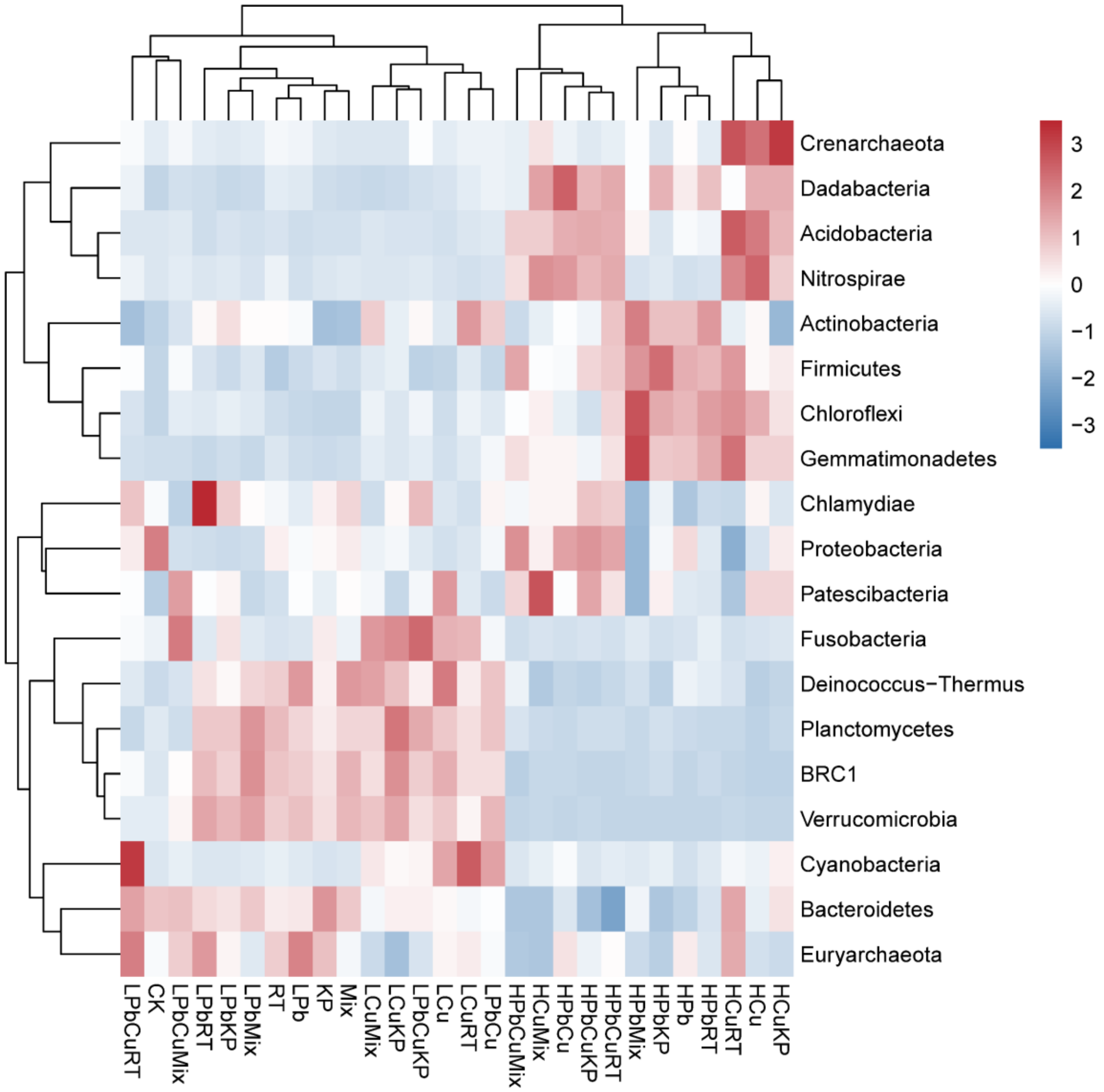

Figure S3 The heatmap of beta diversity estimates of soil bacteria at phylum level based on weighted UniFrac distances. Abbreviations have the same meanings as presented in Figure S1.

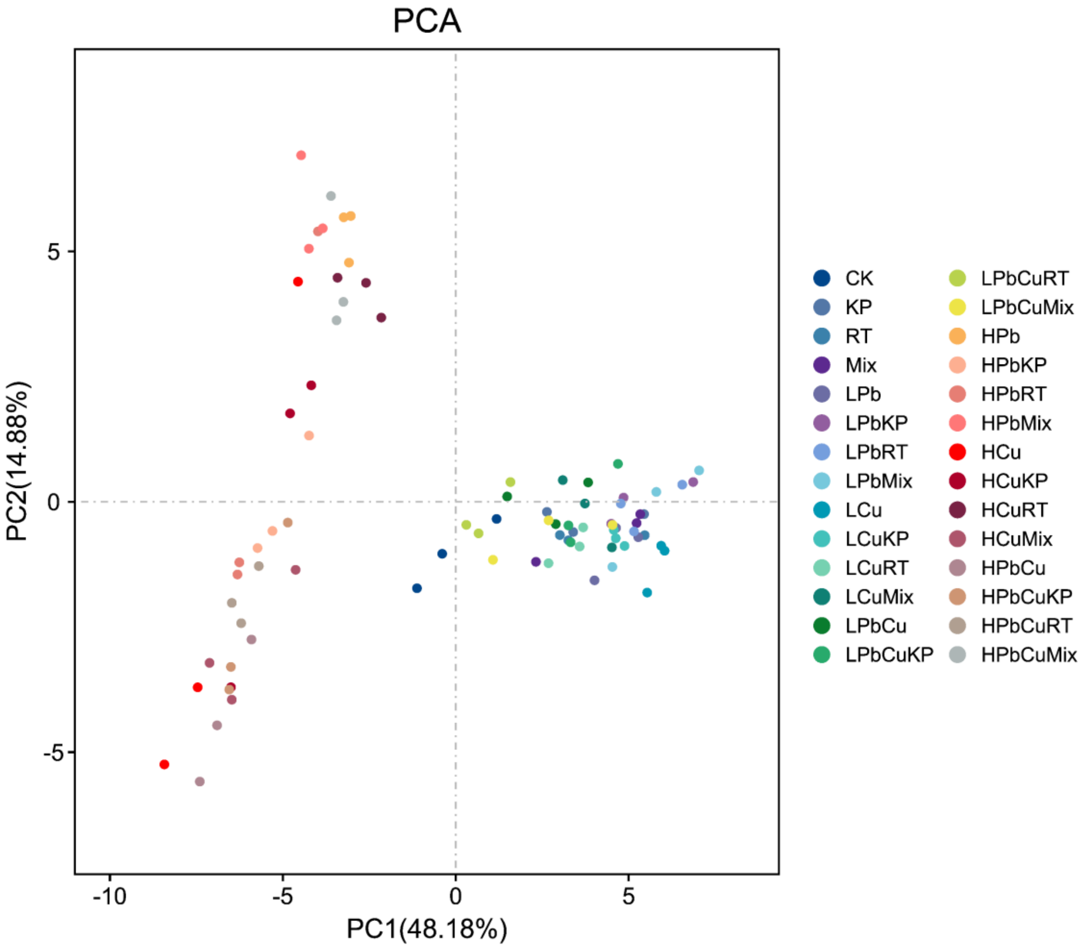

Figure S4 PCA of soil bacteria based on weighted UniFrac distance. Abbreviations have the same meanings as presented in Figure S1.

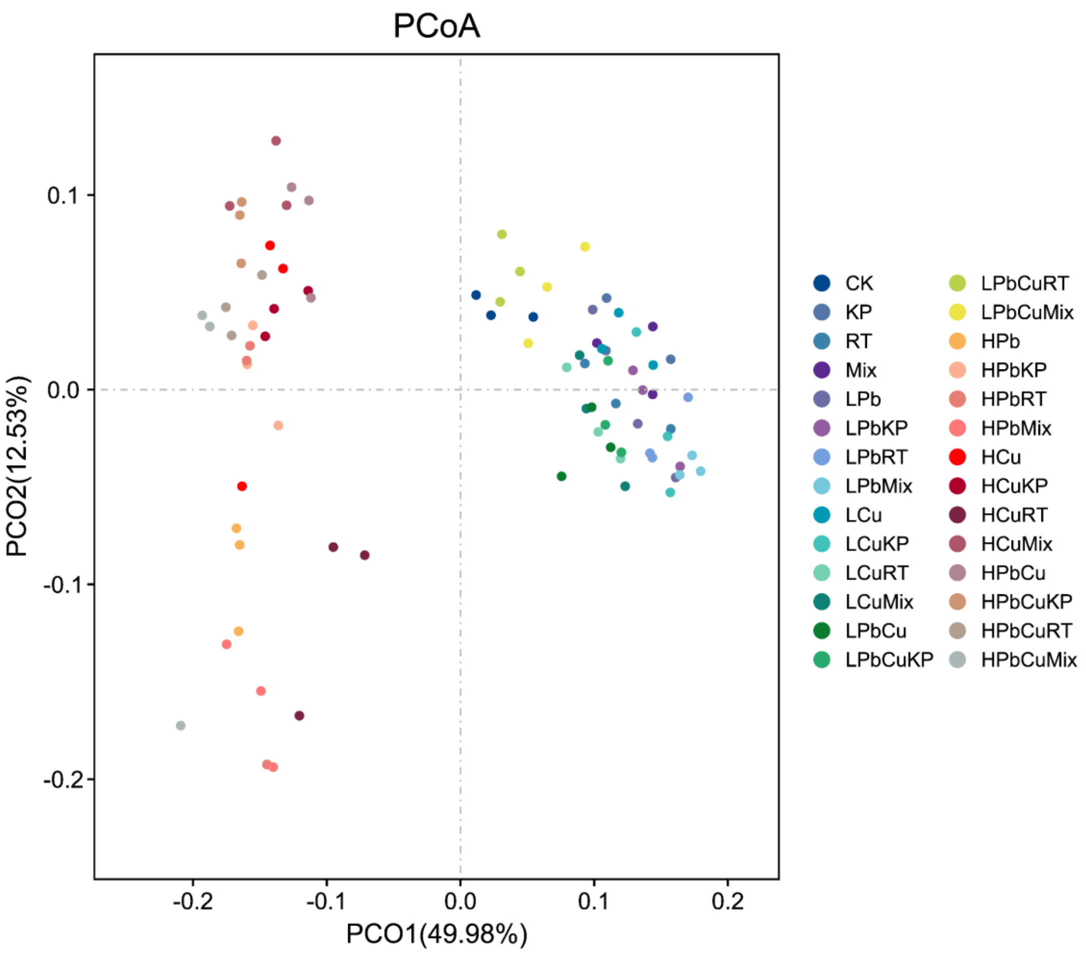

Figure S5 PCoA of soil bacteria based on weighted UniFrac distance. Abbreviations have the same meanings as presented in Figure S1.

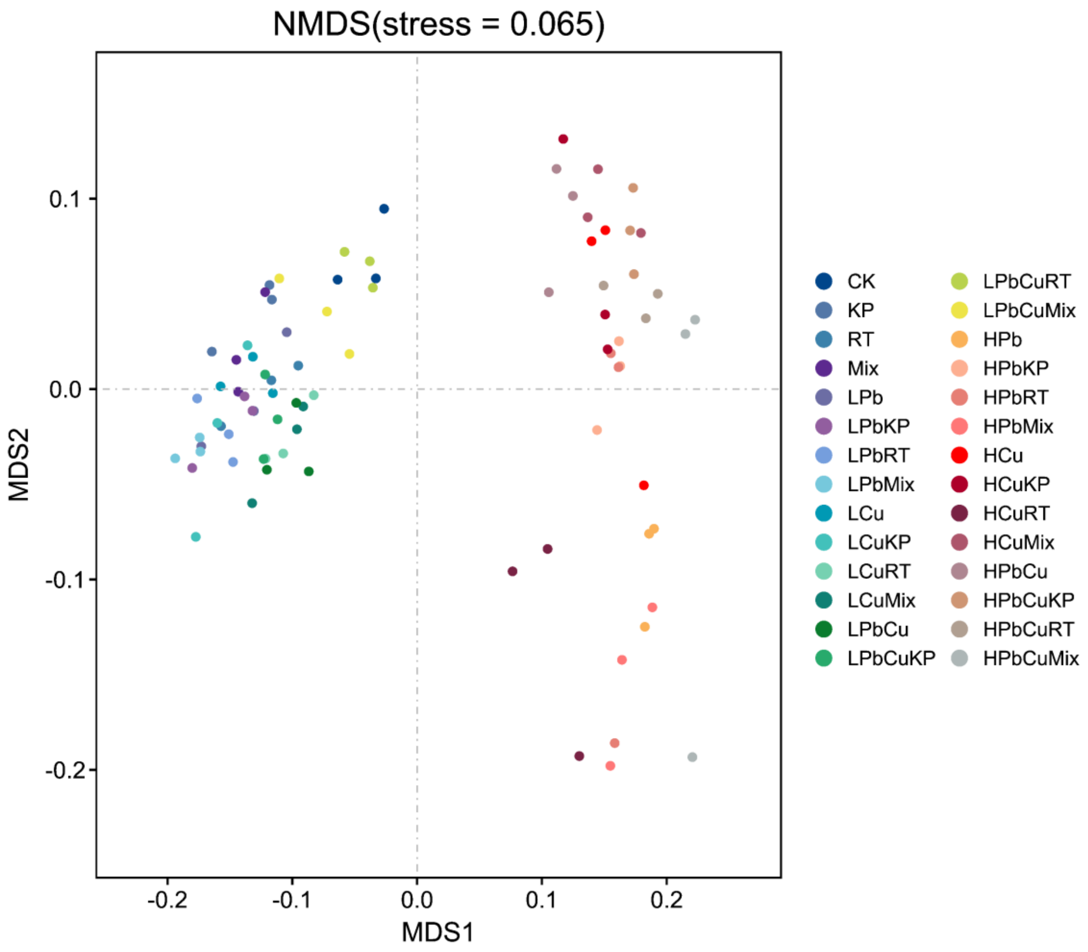

Figure S6 NMDS of soil bacteria based on weighted UniFrac distance. Abbreviations have the same meanings as presented in Figure S1.
